# Impact of Combined Baricitinib and FTI Treatment on Adipogenesis in Hutchinson–Gilford Progeria Syndrome and Other Lipodystrophic Laminopathies

**DOI:** 10.3390/cells12101350

**Published:** 2023-05-09

**Authors:** Ramona Hartinger, Eva-Maria Lederer, Elisa Schena, Giovanna Lattanzi, Karima Djabali

**Affiliations:** 1 Epigenetics of Aging, Department of Dermatology and Allergy, TUM School of Medicine, Munich Institute of Biomedical Engineering (MIBE), Technical University of Munich (TUM), 85748 Garching, Germany; ramona.hartinger@tum.de (R.H.); eva.lederer@tum.de (E.-M.L.); 2 Unit of Bologna, CNR Institute of Molecular Genetics “Luigi Luca Cavalli-Sforza”, 40136 Bologna, Italygiovanna.lattanzi@cnr.it (G.L.); 3 IRCCS Istituto Ortopedico Rizzoli, 40136 Bologna, Italy

**Keywords:** progerin, lonafarnib, baricitinib, lamin A, adipogenesis, progeria, lipodystrophy, JAK/STAT, skin derived precursors, laminopathies

## Abstract

Hutchinson–Gilford progeria syndrome (HGPS) is a rare genetic disease that causes premature aging symptoms, such as vascular diseases, lipodystrophy, loss of bone mineral density, and alopecia. HGPS is mostly linked to a heterozygous and de novo mutation in the LMNA gene (c.1824 C > T; p.G608G), resulting in the production of a truncated prelamin A protein called “progerin”. Progerin accumulation causes nuclear dysfunction, premature senescence, and apoptosis. Here, we examined the effects of baricitinib (Bar), an FDA-approved JAK/STAT inhibitor, and a combination of Bar and lonafarnib (FTI) treatment on adipogenesis using skin-derived precursors (SKPs). We analyzed the effect of these treatments on the differentiation potential of SKPs isolated from pre-established human primary fibroblast cultures. Compared to mock-treated HGPS SKPs, Bar and Bar + FTI treatments improved the differentiation of HGPS SKPs into adipocytes and lipid droplet formation. Similarly, Bar and Bar + FTI treatments improved the differentiation of SKPs derived from patients with two other lipodystrophic diseases: familial partial lipodystrophy type 2 (FPLD2) and mandibuloacral dysplasia type B (MADB). Overall, the results show that Bar treatment improves adipogenesis and lipid droplet formation in HGPS, FPLD2, and MADB, indicating that Bar + FTI treatment might further ameliorate HGPS pathologies compared to lonafarnib treatment alone.

## 1. Introduction

Hutchinson–Gilford progeria syndrome (HGPS; OMIM #176670) is a rare genetic disease with similar symptoms to physiological aging, including vascular disease, subcutaneous fat loss, sclerodermatous skin, loss of bone mineral density, and hair loss [1,2,3,4]. HGPS affects one child in 4–8 million births worldwide [2]. In 2022, approximately 140 children and young adults with HGPS were reported worldwide, with an average life expectancy estimated at 14.5 years [3,5]. Cardiovascular diseases are the major cause of HGPS mortality [2,6]. HGPS is primarily caused by a heterozygous single-point de novo mutation in the lamin A (LMNA) (c.1824 C > T; p.G608G), resulting in a cryptic splice site in exon 11 and the loss of 50 amino acids at the C-terminus of lamin A. The truncated prelamin A protein is known as progerin. Wild-type prelamin A undergoes several specific posttranslational modifications to form mature lamin A, including farnesylation of the C-terminal cysteine, cleavage of the last three amino acids, and carboxymethylation of the C-terminal cysteine, followed by a second upstream cleavage [6,7]. In HGPS, the final upstream cleavage step is not possible because the cleavage site for ZMPSTE24 is missing, generating a permanently farnesylated mutant prelamin A (progerin) [6,8,9]. Progerin is abnormally incorporated into the nuclear envelope as it remains farnesylated. Consequently, progerin accumulation in HGPS nuclei causes cytotoxicity in cells including changes in the nuclear lamina, nuclear disorganization and malfunction, premature senescence, and cell death [1,8,9,10].

Lipodystrophy is characterized by a general or selective loss of subcutaneous and visceral fat and alteration in the body fat compositions [11,12]. Genetic defects, causing lipodystrophy, can directly affect the differentiation of adipocytes, the lipid droplet formation, or the triglyceride transport [12,13]. There are three types of adipose tissues in humans: white (WAT), brown (BAT), and beige. BAT cells contain a large number of mitochondria and are localized in visceral tissues. BAT is involved in thermoregulation during adaptive thermogenesis [14,15]. The beige adipose tissue consists of brown-like adipocytes distributed in WAT [16] and is also involved in thermogenesis by absorbing large amounts of glucose. Beige adipose tissue can be found in muscles [17]. WAT constitutes the largest proportion of body fat, and its primary function is energy storage to regulate the energy homeostasis [16,18]. WAT is found throughout the body and is divided into subcutaneous WAT and visceral WAT, with approximately 80–90% of body fat in adults consisting of subcutaneous WAT [19,20,21]. In a healthy state, triglycerides accumulate in WAT and form large fat droplets inside the adipocytes [16,21]. Apart from its fat storage capacity, adipose tissues play an important hormonal and metabolic regulatory roles [22], and their dysfunction or absence can cause metabolic diseases, steatohepatitis and hepatic cirrhosis, premature cardiovascular disorders, and organ failure [12,13,22].

Lipodystrophy is a prominent clinical feature in patients with HGPS, with 20% of HGPS patients showing generalized lipodystrophy as the first predominant symptom, which can start as early as at six months of age [23]. Adipose tissues first decrease in the limbs and the thorax, then in the neurocranium, and later in the facial, buccal, and pubic areas. In most HGPS cases, abdominal fat remains unaffected, which confers a dominant abdomen characteristic in children with HGPS. Owing to thinning of the skin and the loss of subcutaneous adipose tissue, children with HGPS are characterized by prominent eyes and visible blood vessels on the face and scalp [2,3,23]. Interestingly, lipodystrophy also occurs in other laminophaties such as familiar partial lipodystrophy type 2 (FPLD2) and mandibuloacral dysplasia type B (MADB) [13,24,25]. Patients with HGPS, FPLD2, or MADB harbor mutations on the LMNA or ZMPSTE24, causing abnormalities in lamin processing and cellular changes. Overall, these diseases show typical lipodystrophy symptoms, such as subcutaneous fat loss and metabolic alterations [2,6,24,25,26,27].

FPLD2 is an autosomal dominant genetic disorder caused by a mutation in the LMNA that encodes lamin A and lamin C [13,22,24]. More than 80% of FPLD2 patients carry a single point mutation at position R482 located in the immunoglobulin fold domain of lamin A [26]. Usually, the first symptoms begin during puberty and manifest as an atypical distribution of subcutaneous fat (limbs, trunk, and extremities) and an accumulation of adipose tissue (neck, face, and back). Moreover, symptoms such as cardiovascular diseases, insulin resistance, hypertriglyceridemia, liver diseases, atherosclerosis, and altered bone formation have been reported [13,22,24,26,28]. Previous studies have reported a decrease in pre-adipocyte differentiation and adipogenic potential, impaired lipid droplet formation, and reduced autophagy in patients with FPLD2 [24,26,29,30].

MADB is a rare premature aging syndrome caused by a heterozygous mutation in the ZMPSTE24 gene on chromosome 1p34 encoding the enzyme zinc metalloprotease ZMPSTE24 [25]. ZMPSTE24 C-terminally cleaves the farnesylated and carboxymethylated tail of prelamin A to produce the mature lamin A [25,31]. In MADB, mutations in ZMPSTE24 causes a decrease or absence in the catalytic activity of this enzyme leading to prelamin A nuclear accumulation. MADB patients suffer from osteoporosis, fat loss (lipodystrophy type B), metabolic abnormalities, insulin resistance, delayed growth, dental crowding, skin atrophy, and brittle hair [25,26,27,31].

Although FPLD2, HGPS, and MADB carry different mutations, they are characterized by the expression of abnormal lamin A and the accumulation of permanently farnesylated prelamin A or progerin. All three diseases exhibit symptoms associated with lipodystrophy and altered adipogenesis. At the cellular level, toxic progerin or prelamin A accumulation causes DNA damage, nuclear dysfunctions, altered gene expression, and metabolic defects, which drive cells towards premature senescence and apoptosis [12,32,33,34,35]. Senescent cells remain in a state of irreversible permanent cell cycle arrest and produce a bioactive secretome, known as the senescence-associated secretory phenotype (SASP) [36,37]. The SASP acts as a primary mediator in senescent cells, and secreted inflammatory factors and proteases communicate with the microenvironment and the immune system [37,38]. SASP paracrine signaling has negative effects, including modulation of numerous pathways, such as ROS, MAPK signaling, proliferation, and WNT signaling [39], which can cause chronic and low-grade inflammation due to the constitutive activation of immune cells and secretion of proinflammatory cytokines [36,37,40].

Squarzoni et al. (2021) showed that progerin or farnesylated prelamin A induced the activation of NF-κB and interleukin 6 (IL) promoters and the increased of IL-6 levels in HGPS and MADB fibroblasts [41]. In vivo studies on LmnaG609G/G609G progeroid mice demonstrated that the inhibition of IL-6 with tocilizumab, a neutralizing antibody against IL-6 receptors, caused a decrease in senescence and progerin levels and ameliorated nuclear defects [41]. Additionally, prelamin A or progerin accumulation induced the activation of a NF-κB-driven inflammation via ATM and NEMO in ZMSPTE24-deficient and LmnaG609G mice, resulting in nuclear envelope defects and progeroid symptoms [34]. Adipose tissue appears to be highly sensitive to progerin accumulation [42]. For instance, progerin accumulation and high paracrine activation in adipocyte tissue caused chronic inflammation and cellular senescence in a LmnaG609G/G609G mouse model [42]. Additionally, loss of fat and other fat deposits was observed in LmnaG609G/G609G mice [43,44].

Over the years, several strategies have been investigated to treat HGPS. Targeting the post-translational modification of progerin to prevent its tethering to the nuclear envelope and increase its clearance is a promising strategy. The first tested compound is a farnesyltransferase inhibitor (FTI, lonafarnib) [6,45], and studies have shown that FTI ameliorates several cellular phenotypic changes in HGPS [10,45,46,47]. Clinical trials with lonafarnib caused a decrease in the mortality rate and improved bone mineralization, weight, and cardiovascular systems in patients with HGPS [1,6,45]. Presently, lonafarnib has been approved by the FDA for the treatment of HGPS [48]. Although lonafarnib ameliorates HGPS children’s condition, it is not a cure, and new therapies are urgently needed. One novel potential strategy is to reduce the downstream toxic effect of progerin at the cellular level. Recent studies have demonstrated that the JAK-STAT signaling is overactivated in HGPS cells, and that chronic low-grade inflammation may be a common etiology of various pathologies affecting patients with HGPS [49,50]. The JAK1/2-STAT1/3 inhibitor baricitinib (Bar), an FDA-approved treatment for rheumatic arthritis [51], has been shown to reduce senescence and progerin levels and improve nuclear shape, proliferation, and mitochondrial functions [50]. Moreover, several studies have shown a potential link between adipogenesis and the JAK/STAT pathway [52,53,54,55,56]. The JAK-STAT pathway can influence the proliferation and function of mature adipocytes and modulate their tissues [53,55]. Therefore, these findings suggest that Bar treatment may improve adipogenesis in HGPS.

As farnesylated progerin and farnesylated prelamin A expression induce several cellular changes, including premature senescence, it is likely that JAK-STAT overactivation may also occur in FPLD2 and MADB cells. Here, we elucidate the role of JAK/STAT signaling in the development of lipodystrophy in HGPS, FPLD2, and MADB, using an in vitro adipogenesis model. Specifically, we examined the effect of combined treatment with Bar and lonafarnib on adipogenesis in cells derived from patients with HGPS, FPLD2, and MADB. An ex vivo cellular model consisting of skin-derived precursors (SKPs) isolated from human primary fibroblast HGPS, FPLD2, and MADB was established using the pH-stress method [57,58]. Multipotent SKPs are found in adult human skin and express stem cell markers [59,60,61]. The SKPs were differentiated into adipocytes and cultured with Bar and/or FTI.

## 2. Materials and Methods

### 2.1. Cell Culture

In this study, the following human primary dermal fibroblast cell lines were used: control cell strains GM05757C (7-year-old male), GM05567A (12-year-old male), and GM01651C (13-year-old female) without mutations; HGPS cell strains HGADFN003 (2-year-old male), HGADFN164 (4-year-old female), and HGADFN178 (6-year-old female) with mutation on LMNA Exon 11, heterozygous c.1824C > T (p.Gly608Gly); FPLD2 cell strains CCLMA00336s, CCLMS337s, and CCBB00466s with mutations on position LMNA R482Q; MADB cells PSADFN317 (3-year-old male) and PSADFN318 (5-month-old male) with mutation on ZMPste24 Exon 6, heterozygous c.743C > T (p.Pro248Leu); Exon 10, heterozygous c.1349G > A. Human normal primary dermal fibroblast cells were obtained from the Coriell Institute for Medical Research (Camden, NJ, USA). HGPS and MADB cells were obtained from the Progeria Research Foundation Cell and Tissue Bank, and FPLD2 cells were provided by the Institute of Molecular Genetics IGM Bologna (G. Lattanzi).

The fibroblast monocultures were cultured in DMEM (Thermo Fisher—Gibco, Waltham, MA, USA, D6429) supplemented with 15% fetal bovine serum (FBS; Thermo Fisher—Gibco, 10270106), 1% L-glutamine (Thermo Fisher—Gibco, 25030081), 1% gentamycin (Thermo Fisher—Gibco, 15710049), and 1% penicillin/streptomycin (Thermo Fisher—Gibco, 1514022). All fibroblasts were cultured in a cell incubator (Binder, Tuttlingen, Germany, 9140-0046) at 37 °C and under a 5% CO_2_ atmosphere. The monocultures were sub-cultured and used with at 80% confluence, and a senescence < 5%. Additionally, monocultures with senescence >20% were used for western blots and immunofluorescence.

### 2.2. Senescence Associated βeta-Galactosidase Assay

β-galactosidase assay was performed to assess cell senescence, as previously described by Dimri et al. (1995) [62]. The adherent cells were washed once with phosphate-buffered saline (PBS) and fixed for 5 min in 0.2% glutaraldehyde (Sigma-Aldrich, St. Louis, MO, USA, G5882), and 2% formaldehyde (Sigma-Aldrich, 104003). The fibroblasts were washed 2 times with PBS and incubated overnight at 37 °C (in absence of CO_2_) with SA-β-Gal staining solution (5 mM potassium ferricyanide (Merck KGaA, 104973, Darmstadt, Germany), 5 mM potassium ferrocyanide (Sigma-Aldrich, P9387), 2 mM MgCl_2_ (Sigma-Aldrich, M-1028), 150 mM NaCl (Sigma-Aldrich, 310166), 0.5 mg/mL 5-bromo4-chloro-3-indolyl P3-D-galactoside (X-gal) (Sigma-Aldrich, 3117073001), and 40 mM citrate/sodium phosphate buffer (pH 6.0) at 37 °C). An average of 1000 cells were counted per sample, and blue-stained cells were classified as senescent.

### 2.3. Western Blot

Fibroblasts were washed with PBS and collected by trypsinization using trypsin-EDTA (Thermo Fisher—Gibco, 25200056), pelleted by centrifugation at 350× *g* for 5 min at room temperature (RT), and lysed (150 mM NaCl, 1% Triton, 1% SDS, 1 mM EDTA, 50 mM Tris). Total protein concentration was determined using the Bradford assay, with BSA as a standard (BioRad Laboratories, 5000206, Hercules, CA, USA). Proteins (10 µg) were separated in an 8% gel via electrophoresis and transferred onto nitrocellulose membranes via wet-transfer. The membranes were blocked by incubating with 5% non-fat milk for 1 h, followed by incubation with the primary antibodies, including anti-prelamin A (Merck Millipore, 7G11, rat, 1:2000, overnight, Dallas, TX, USA), anti-lamin A/C (E1, sc-376248, Santa Cruz Biotechnology, 1:10000), anti-lamin B1 (C12, sc-365214, Santa Cruz Biotechnology, 1:5000), and anti-GAPDH (G9545, Sigma-Aldrich, 1:5000) overnight at 4 °C. After washing three times with TBS-Tween, the membranes were incubated with horseradish peroxidase-conjugated secondary antibodies (Jackson ImmunoResearch Laboratories, West Grove, PA, USA), including anti-rabbit (1:5000), anti-rat (1:5000), or anti-mouse (1:5000) for 1 h at RT. Thereafter, luminol-enhanced chemiluminescence was performed and the signals were visualized using ChemiDoc™ MP and quantified using ImageJ software (NIH). The nlots were quantified by normalizing to GAPDH (internal control) expression levels.

### 2.4. Low-pH SKP Isolation Method and Culture of SKPs

SKPs were isolated from primary fibroblast cultures using the low pH stress method. Briefly, primary fibroblast cultures with senescence <5% and at 80% confluency were used for this analysis. Briefly, fibroblast cultures were washed with PBS, collected using trypsin-EDTA (Thermo Fisher—Gibco, 25200056), pelleted by centrifugation at 350× *g* for 5 min at RT, and washed with PBS. For SKP isolation, cells (1 × 10^6^) were resuspended in pH-adjusted Hank’s balanced salt solution (HBSS) buffer (Thermo Fisher—Gibco, 14175053). The pH of the HBSS buffer was adjusted to 5.7 using HCL (Merck KGaA, Darmstadt, Germany, 1.00319.2500), and the cells were incubated at 37 °C for 25 min and resuspended every 5 min. After 25 min of incubation, the cell suspension was centrifuged at 350× *g* for 5 min at RT, and the cell pellet was suspended in 6 mL of SKP media (4:1—DMEM low glucose (Thermo Fisher—Gibco, 21885025):F12 (Thermo Fisher—Gibco, 21765029), 20 ng/mL EGF (Thermo Fisher—Gibco, PHG0311), 40 ng/mL bFGF (Thermo Fisher—Gibco, PHG0026), 2% *v*/*v* B27 (Thermo Fisher—Gibco, 17504044), 0.5 g/mL fungizone (Thermo Fisher—Gibco, 15290018), and 100 U/100 _g/mL penicillin/streptomycin) and equally split in two T25 non-tissue-culture-treated flasks (Fisher Scientific—Falcon, Hampton, NH, USA, 10112732) (Budel und Djabali, 2017). The SKP cultures were resuspended daily to prevent adherence of the SKP spheroids to the plastic flask. SKP cultures were supplemented every 2 d with 10× SKP media (10× concentration of EGF, bFGF, and B27), which was diluted to a final concentration of 1× SKP media.

### 2.5. SKP Cell Differentiation into Adipocytes

At 4 d after cultivation, the SKPs were collected and centrifuged at 350× *g* for 5 min at RT. The spheroids were washed twice with PBS, dissociated using trypsin-EDTA (Thermo Fisher—Gibco, 25200056), and seeded onto cover slips in 24-well plates. The seeding density differed for each cell type. Control SKPs were seeded at 8 × 10^5^ cells per well, and HGPS SKPs were seeded at 1.2 × 10^6^. The cells were cultured in adipocyte differentiation media (ADM) consisting of DMEM supplemented with 4.5 g/L glucose (Thermo Fisher—Gibco, 21885025), 0.5 mM 3-isobutyl-1-methylxanthine (IBMX, Sigma-Aldrich, St., Louis, MO, USA, I7018, stock in absolute ethanol (VWR chemicals, Radnor, PA, USA, 20821.33)), 10 μg/mL insulin (Sigma-Aldrich, I2643, stock in 0.01 M HCL [Merck KGaA, 1.00319.2500] in Ultra-Pure water from MilliQ [MQ]), 100 μM L-Ascorbic Acid (Sigma-Aldrich, A8960, stock in Ultra-Pure water from MilliQ [MQ]), 1 μM dexamethasone (Sigma-Aldrich, D4902 (stock in absolute ethanol)), 10% FBS, 0.5 μg/mL fungizone, 50 μM indomethacin (Sigma-Aldrich, I7378, stock in 100% DMSO [Sigma-Aldrich, D2650]), and 100 U/100 μg/mL penicillin/streptomycin. The media was replaced every 2–3 d [58].

For the drug treatment, a mock solution (no drug), 1 μM baricitinib (Selleck Chemicals GmbH, Munich), 0.025 μM lonafarnib (Merk KGaA, Darmstadt, Germany), or a combination of 1 μM of baricitinib and 0.025 μM of lonafarnib (Bar + FTI) was added to ADM.

### 2.6. Oil Red O (ORO) Staining

Differentiated adipocytes were fixed in 4% paraformaldehyde (PFA; Merck KGaA, 104005) for 30 min. Next, the cells were incubated in 60% isopropanol for 5 min, followed by incubation in ORO staining solution for 5 min. Thereafter, the coverslips were washed in tap water and screened under a microscope. The staining solution was prepared by mixing three parts of ORO stock solution (ORO powder (Sigma-Aldrich, O0625) in 99% isopropanol) with two parts of demineralized water and filtering two times using a filter paper (Rotilabo-Rundfilter, Typ 11A, Carl Roth GmbH + Co. KG, Karlsruhe, Germany).

### 2.7. Bodipy Staining

The differentiated adipocytes were fixed in 2% PFA (Merck KGaA, 104005) for 20 min and washed once with PBS. Lipid droplets were stained with 2 μM of Bodipy (Invitrogen, Waltham, MA, USA, D3922) for 45 min and then washed three times with PBS. The cells were counterstained with DAPI Vectashield mounting medium (Vector Laboratories, Burlingame, CA, USA, VEC-H-1200), and images were captured using an Axio Imager D2 fluorescence microscope (Light source: X-cite 120Q (EXFO Photonic Solutions Inc., Mississauga, ON, Canada); objectives used: EC-Plan Neofluar 10×/0.3 (420340-9901, Carl Zeiss), Plan-Apochromat 40×/0.95 Korr (440654-9902, Carl Zeiss); camera used: AxioCam MRm (Carl Zeiss, Oberkochen, Germany); excitation and emission filters used: filter set 49 (424931, Zeiss), filter set 38 HE (424931, Zeiss)).

### 2.8. Immunocytochemistry

Adipocytes, grown on glass cover slips, were fixed with 2% PFA (Merck KGaA, 104005) for 10 min and washed 3 times for 5 min with PBS. The cells were permeabilized with 0.2% Triton X-100 in PBS for 5 min and washed once with PBS for 5 min. After permeabilization, the cells were blocked with 10% FBS (Thermo Fisher—Gibco, 10270106) in PBS for 30 min at RT, then incubated overnight at 4 °C with the following primary antibodies: rat anti prelamin A (Merk Millipore, 7G11, 1:400, overnight), mouse anti-Lamin B1 (Santa Cruz Biotechnology, 1:200, overnight), and rabbit anti-progerin [63]. After four washes with blocking buffer, the cells were incubated with the secondary antibodies: affinity-purified Alexa Fluor^®^ 488 or 555 conjugated anti-rat/-rabbit/-mouse antibodies (Life Technologies, Carlsbad, CA, USA, A21202 anti-mouse-488, A21208 anti-rat-488, and A31572 anti-rabbit-555, 1:1000) for 1 h at RT. Thereafter, the cells were washed twice with blocking buffer and twice with PBS and counterstained with DAPI Vectashield mounting medium (Vector Laboratories, Burlingame, CA, USA, VEC-H-1200). Images were captured using an Axio Imager D2 fluorescence microscope (AxioCam MRm, Carl Zeiss, Oberkochen, Germany).

### 2.9. Image Analysis

Images were analyzed using Fiji software (ImageJ 1.53f51, Java 1.8.0_172, Wayne Rasband, and contributors to the National Institutes of Health, USA). Brightness and contrast were adjusted [64], and ORO intensity, lipid droplet (LD) size, BODIPY intensity, and BODIPY-positive cells were determined. Inkscape (Version 1.1.2 (b8e25be833, 2022-02-05), GPL) was used for illustration. The total area of BODIPY was quantified by measuring the area with BODIPY-positive signal compared to total area of the coverslip.

### 2.10. Statistical Evaluation and Graphics

Three biological replicates were analyzed for each cell strain. For senescence, dysmorphic nuclei and BODIPY-positive cells (1000 cells per cell strain) were counted under the various treatment conditions. The lipid droplet size was measured using 150 cells/cell strain and treatments.

All results are presented as mean ± SD and were generated using the student’s t-test to compare the difference between 2 groups. For multiple groups’ comparison, 2-way ANOVA was used. Calculations and graphs were generated using GraphPad Prism (Version 6.01, GraphPad, San Diego, CA, USA). The following symbols indicate statistical significance: ns, not significant (*p* > 0.05); * *p* ≤ 0.05; ** *p* ≤ 0.01; and *** *p* ≤ 0.001.

## 3. Results

### 3.1. Adipocyte Differentiation of HGPS SKPs in the Presence of FTI and Baricitinib

**Figure 1:**
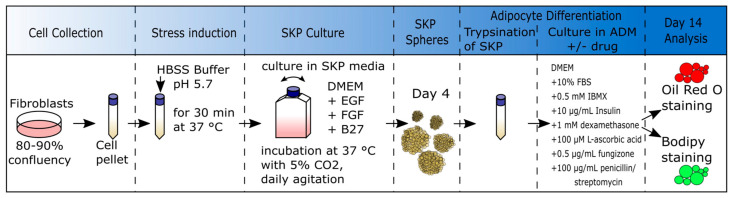
Schematic representation of low-pH isolation of SKPs from primary fibroblast cultures. Young fibroblasts (control cells passages 16–21, HGPS cells passages 10–16, FPLD2 cells passages 9–14, MADB cells passages 12–13) were treated with HBSS Buffer (pH 5.7) for 30 min at 37 °C. The SKPs were cultured in SKP media (DMEM low glucose plus EGF, FGF, and B27). After 4 d, SKPs spheroids were trypsinated and seeded in ADM (DMEM plus 10% FBS, IBMX, insulin, dexamethasone, L-ascorbic acid, fungizone, and penicillin/streptomycin) with or without drugs. After 14 d of differentiation, cells were fixed and stained with Oil Red O or BODIPY staining. HBSS: Hank’s Balanced Salt Solution; ADM: adipocyte differentiation media; DMEM: Dulbecco´s modified Eagle medium; EGF: epidermal growth factor; FGF: fibroblast growth factor; SKP´s: skin-derived precursor cells; FBS: fetal bovine serum; IBMX: isobutylmethylxanthine.

Previous studies have successfully isolated SKP spheroids from primary fibroblast cultures using the low-pH stress method [57]. Senescence plays an important role in SKP differentiation [58]; therefore, young fibroblast cultures with <5% senescence rate were used for the analysis to prevent the effect of age on the differentiation potential of SKPs. The SKP isolation method is illustrated in Figure 1. After low-pH stress isolation, the SKPs were cultured in SKP medium, dissociated after 4 d (Figure 1), and cultured in adipocyte differentiation media (ADM) supplemented with either 0.025 μM FTI, 1 μM Bar, or the combination of 0.025 μM FTI and 1 μM Bar, or vehicle for 14 d to examine the effect of lonafarnib (FTI) and the JAK 1/2 inhibitor Bar on adipocyte differentiation

**Figure 2:**
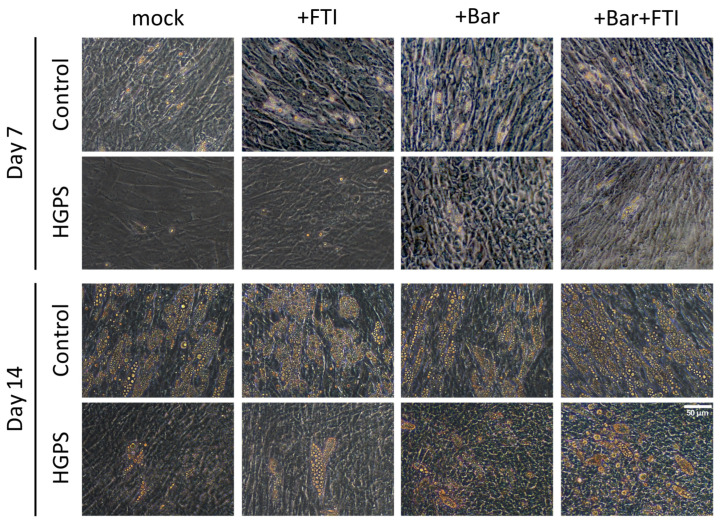
Bright-field imaging of control (GM05567A, GM05757C, GM01651C) and HGPS (HGADFN003, HGADFN164, HGADFN178) adipocytes treated with the blank (mock), 0.025 μM FTI, with 1 μM baricitinib and a combination of 0.025 μM FTI and 1 μM baricitinib at 7 and 14 d after culture in adipocyte differentiation medium (ADM). SKPs were isolated with the low-pH stress method from primary fibroblast cultures and grown in SKP media. At 4 d, SKP were dissociated and cultured in ADM with indicated treatments. Lipid accumulation was clearly observed in control and HGPS adipocytes at 14 d of differentiation. Increased lipid droplet formation was observed in HGPS adipocytes treated with Bar and Bar + FTI. Scale bar: 50 μm.

Lipid droplets were visible in both control and HGPS cells at 7 d after differentiation; however, mock- and FTI-treated HGPS cells had lower number of droplets compared to control groups (Figure 2, panel, day 7). Additionally, lipid formation was not affected by the different treatments in normal cells, whereas Bar and Bar + FTI treatments increased lipid droplet accumulation and adipocyte differentiation in HGPS cells compared to mock- or FTI-treated HGPS cells (Figure 2). After 14 d, there was a remarkable increase in the accumulation of lipid droplets in the control and HGPS cells (Figure 2). Specifically, lipid droplet accumulation and adipocyte differentiation were significantly lower in untreated and FTI-treated HGPS cells compared to Bar- or Bar + FTI-treated HGPS cells (Figure 2, panel, day 14). Collectively, these results indicated that Bar and Bar + FTI treatment improved the differentiation of HGPS-derived SKPs into adipocytes.

### 3.2. Baricitinib Alone or in Combination with FTI Improve Adipogenesis of HGPS SKPs

**Figure 3:**
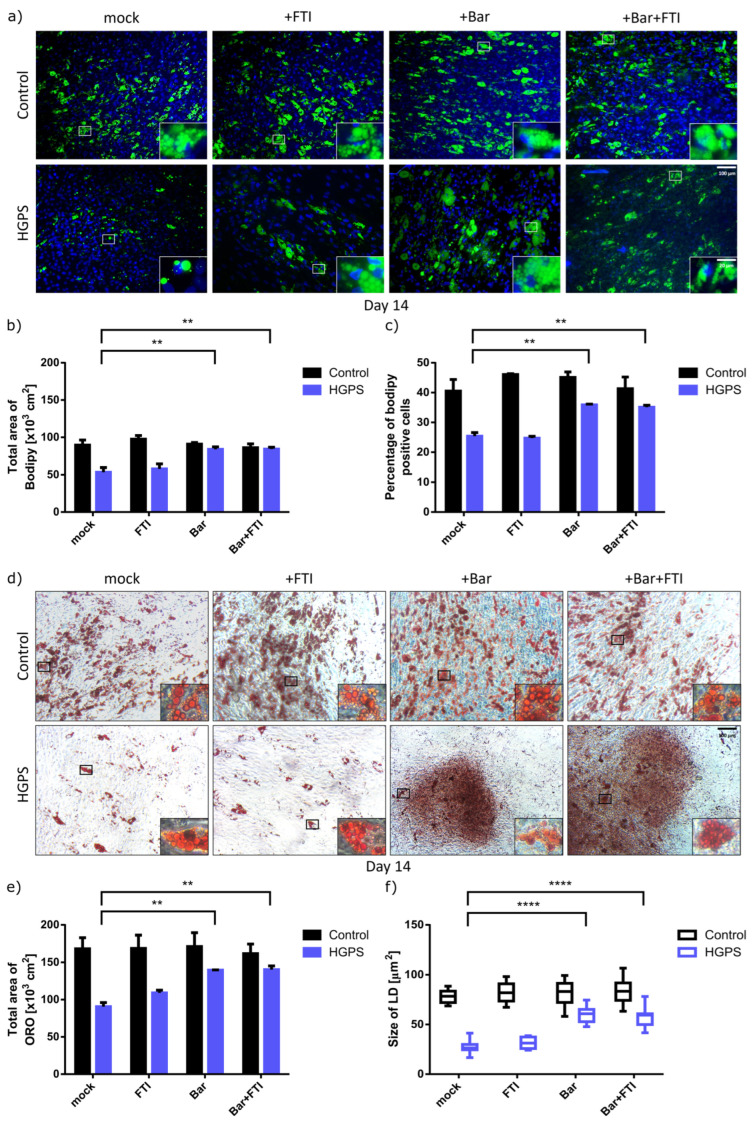
BODIPY (green) and oil red O (ORO) staining (red) of control and HGPS adipocytes after 14 d of differentiation under different treatment conditions. Treatments: no drug (mock), with 0.025 μM FTI, with 1 μM baricitinib and a combination of 0.025 μM FTI and 1 μM baricitinib. (**a**) BODIPY staining of lipid vesicles. Representative images for control (GM05567A, GM05757C, GM01651C) and HGPS (HGADFN003, HGADFN164, HGADFN178) adipocytes at d14 of differentiation. Cells were counterstained with DAPI. Scale bar 100 μm, scale bar: 20 μm. (**b**) Quantification of the total area of BODIPY signal. Total area of BODIPY was quantified by measuring the area with BODIPY-positive signal compared to total area of the coverslip. (**c**) Percentage of BODIPY positive cells. At least 1000 cells were counted per cell strains. (**d**) ORO staining of lipid droplets. Representative images for control (GM05567A, GM05757C, GM01651C) and HGPS (HGADFN003, HGADFN164, HGADFN178) adipocytes. Scale bar 100 μm, total images scale bar: 20 μM (**e**) Quantification of the total area of ORO signal. (**f**) Quantification of the lipid droplet size. (**b**,**c**,**e**,**f**) Values are presented as mean ± SD (*n* = 3); not significant (ns); ** *p* < 0.01; **** *p* < 0.0001; unpaired *t*-test.

To determine whether the treatment with Bar or a combination of Bar + FTI can improve adipocyte differentiation in HGPS, cultures were fixed and stained with ORO or BODIPY at 14 d after differentiation (Figure 3). Adipocyte differentiation efficiency was quantified by analyzing the total area of ORO and BODIPY, measuring the lipid droplet size and counting the BODIPY-positive cells using Fiji software.

Compared with the mock control SKPs, there was no significant difference in the differentiation rate of control SKPs into adipocyte among all treatment regimens, indicating that the drugs did not affect the differentiation of normal (control) SKPs (Figure 3). Specifically, approximately 43% of normal SKPs differentiated into adipocytes and showed a positive BODIPY signal (Figure 3a,c). In contrast, SKPs differentiation into adipocytes was decreased in the mock- and FTI-treated HGPS groups, with only 24% adipocytes and BODIPY positive signal (Figure 3a,c). However, the treatment with Bar or Bar + FTI increased the number of differentiated cells and the accumulation of lipid droplets (Figure 3a–c). Compared with mock-treated HGPS SKPs, BODIPY positive signal increased by 40% in the Bar and Bar + FTI groups, with approximately 35% of the SKPs in Bar and Bar + FTI groups differentiating into adipocytes (Figure 3a,c). Similarly, ORO staining confirmed that Bar or Bar + FTI treatments improved adipocyte differentiation (Figure 3d–f), as evidenced by a 56% increase in the differentiation of Bar- or Bar + FTI-treated HGPS SKPs compared to mock- or FTI-treated HGPS SKPs, reaching a similar differentiation rate as mock-treated control SKPs (Figure 3b,e).

Consistent with the results of BODIPY staining, ORO staining showed that lipid droplet size was not significantly affected by treatments in normal SKPs (Figure 3c,f). In contrast, treatment of HGPS adipocytes with Bar or Bar + FTI increased lipid droplets by 2-fold compared to the mock-treated HGPS group (Figure 3f). Collectively, these results indicated that Bar and Bar + FTI treatments efficiently improved adipogenesis and lipid droplet formation in HGPS SKPs.

### 3.3. Effect of Baricitinib and FTI on FPLD2 and MADB Adipogenesis

Patients affected with FPLD2 and MADB, two other laminopathies linked to lamin A and ZMPSTE24 mutations, respectively, also suffer from lipodystrophy [22,26]. However, FPLD2 and MADB are caused by different mechanistically linked genes and have similar symptoms with HGPS, such as loss of fat and changes in fat depot distribution [13]. Therefore, we examined whether Bar and Bar + FTI treatments can also improve adipogenesis in SKPs isolated from primary fibroblasts derived from these distinct patients.

**Figure 4:**
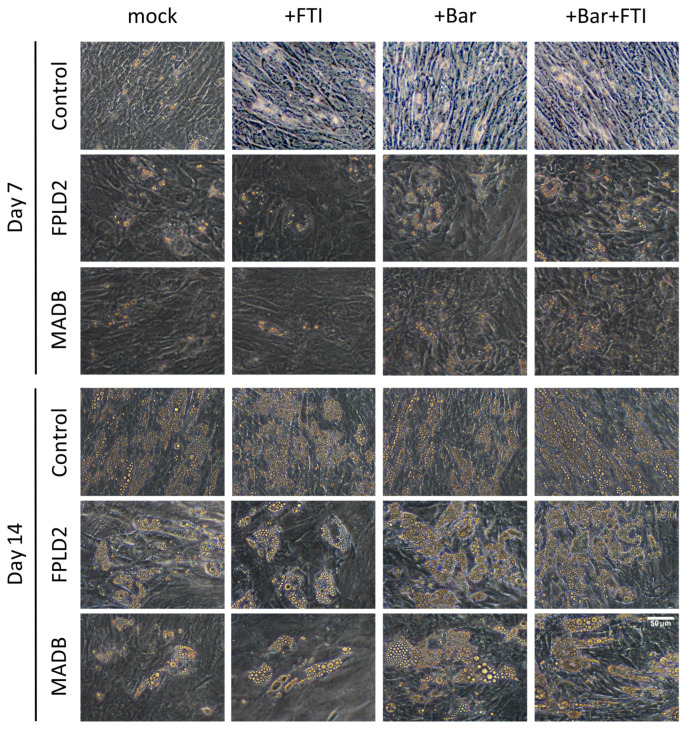
Bright-field imaging of control (GM05567A, GM05757C, GM01651C), FPLD2 (CCLMA00336s, CCLMS337s, CCBB00466s), and MADB (PSADFN317, PSADFN318) adipocytes treated without drug (mock), with 0.025 μM FTI, with 1 μM baricitinib, and with the combination of 0.025 μM FTI plus 1 μM baricitinib at 7 and 14 d after treatments. SKPs were isolated with low-pH stress method from primary fibroblast cultures and grown in SKP media. At 4 d, SKPs were dissociated and cultured in ADM containing indicated regimen. Lipid accumulation was more obvious in control, FPLD2, and MADB adipocytes at 14d of differentiation. Scale bar: 50 μm.

**Figure 5:**
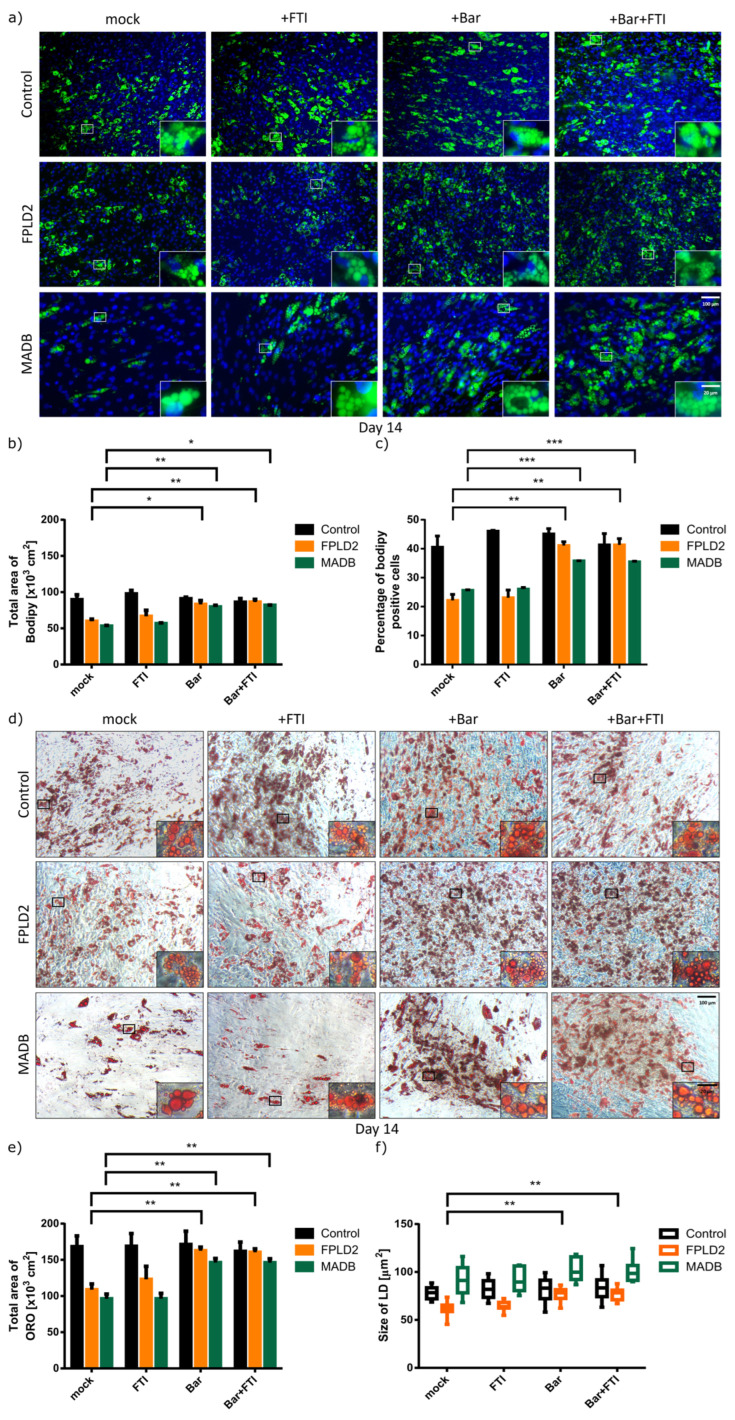
BODIPY (green) and ORO (red) staining of control, FPLD2, and MADB adipocytes treated with 0.025 μM FTI, with 1 μM baricitinib, a combination of 0.025 μM FTI and 1 μM baricitinib, and mock solution after 14d of differentiation. (**a**) BODIPY staining of lipid vesicles. Representative images for control (GM05567A, GM05757C, GM01651C), FPLD2 (CCLMA00336s, CCLMS337s, CCBB00466s), and MADB (PSADFN317, PSADFN318) adipocytes. Cells were counterstained with DAPI. Scale bar 100 μm, total images scale bar: 20 μm. (**b**) Quantification of the total area of BODIPY signal. Total area of BODIPY was quantified by measuring the area of BODIPY-positive signal compared to total area of the coverslip. (**c**) Percentage of BODIPY-positive cells. (**d**) ORO staining of lipid droplets. Representative images for control (GM05567A, GM05757C, GM01651C), FPLD2 (CCLMA00336s, CCLMS337s, CCBB00466s), and MADB (PSADFN317, PSADFN318) adipocytes. Scale bar 100 μm, total images scale bar: 20 μM. (**e**) Quantification of the total area of ORO signal. (**f**) Quantification of the lipid droplet size. (**b**,**c**,**e**,**f**) Values are presented as mean ± SD (*n* = 3); not significant (ns); * *p* < 0.05; ** *p* < 0.01; *** *p* < 0.001; unpaired *t*-test.

SKPs were isolated from young FPLD2 and MADB primary fibroblast cultures (senescence ≤5%) using the low-pH stress method and then differentiated into adipocytes (Figure 1). Adipocyte differentiation was examined and monitored daily. Lipid droplets were observed in control, FPLD2, and MADB groups after 7 d (Figure 4). Control cells were not significantly affected by the different treatments, as a similar number of lipid droplets were observed in all treatment groups (Figure 4). In contrast, mock and FTI treatment caused a decrease in lipid droplet formation in FPLD2 and MADB cells (Figure 4 and Figure 5). However, treatment of FPLD2 and MADB SKPs with Bar and Bar + FTI increased the adipocyte number and lipid droplets formation (Figure 4, panel day 7), which was more obvious after 14 d of treatments (Figure 4 and Figure 5). Notably, Bar- and Bar + FTI-treated FPLD2 and MADB showed higher adipocyte differentiation capability and lipid droplets formation than mock- and FTI-treated cells (Figure 4, panel day 14). Furthermore, MADB adipocytes had larger lipid droplets than control cells at 7–14 d after treatment (Figure 4).

BODIPY staining showed that the adipocyte differentiation rate was 43% in the control cells under all treatment regimens; however, only 22.5 and 30% of FPLD2 and MADB SKPs, respectively, differentiated into adipocytes following mock and FTI treatment, with an obvious decrease in the number of BODIPY-positive cells (Figure 5a,c). In contrast, Bar and Bar + FTI treatments increased the adipocyte differentiation rate by an average of 86% in the FPLD2 and 41% in the MADB groups, respectively (Figure 5a,c), which was confirmed by ORO staining (Figure 5). Similarly, Bar and Bar + FTI treatments significantly increased in adipogenesis and lipid droplets formation in the FPLD2 and MADB groups. Compared to the mock group, Bar and Bar + FTI treatments increased adipocyte differentiation in the FPLD2 and MADB groups by 1.5-fold (Figure 5d,e). Additionally, mock- and FTI-treated FPLD2 adipocytes had smaller-sized lipid droplets compared to the normal (control) cells (Figure 5). In contrast, treatment with Bar and Bar + FTI significantly increased lipid droplet size in the FPLD2 group to a size comparable to that (~76 µm^2^) in the control group (Figure 5f). Lipid droplet size was significantly larger in the MADB adipocytes compared to the control and FPLD2 adipocytes under all treatment regimens; moreover, MADB cell differentiation rate was improved by Bar and Bar + FTI treatments (Figure 5f). Overall, these results indicated that Bar and Bar + FTI improved adipocyte differentiation and lipid droplet formation in both FPLD2 and MADB SKPs.

### 3.4. Lamin Status in HGPS, FFLD2, and MADB Primary Fibroblast Cultures

To further understand how lamin status in HGPS, FPLD2, and MADB primary fibroblast cultures affects the SKP preparation and adipogenic potential, immunocytochemistry and western blot analyses were performed to determine progerin, prelamin A, lamin B1, and lamin A/C expression in young fibroblast cultures (<5% senescence, control cells passages 16–21, HGPS cells passages 10–14, FPLD2 cells passages 9–14, MADB cells passages 12–14) and old fibroblast cultures (>20% senescence, control cells passages 28–31, HGPS cells passages 18–19, FPLD2 cells passages 20–23, MADB cells passages 16–17).

**Figure 6:**
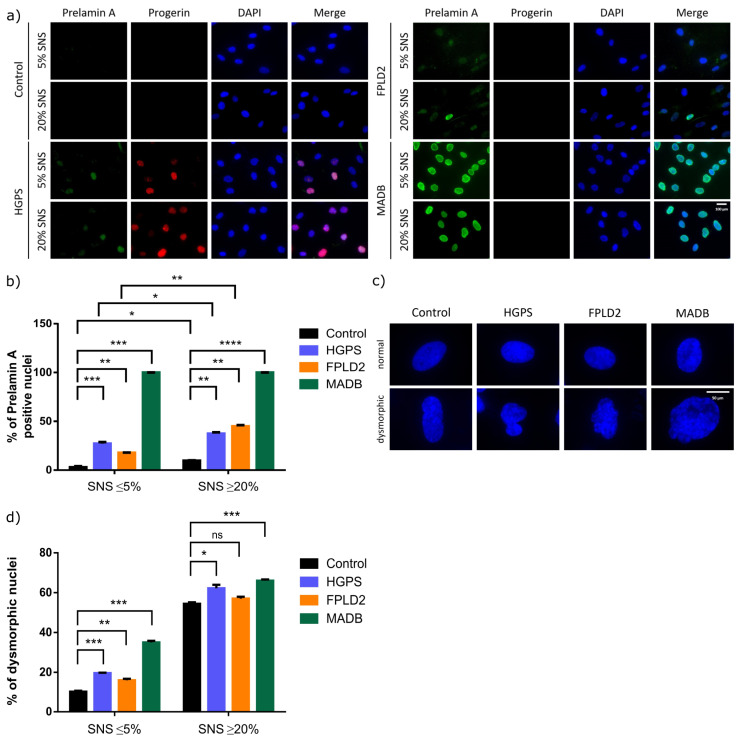
Localization of progerin and prelamin A in fibroblasts derived from different laminopathies: HGPS, FPLD2, and MADB. (**a**) Immunohistochemistry for progerin and prelamin A in young (senescence (SNS) ≤ 5%, control cells passages 16–21, HGPS cells passages 10–14, FPLD2 cells passages 9–14, MADB cells passages 12–14) and old (SNS ≥ 20%, control cells passages 28–31, HGPS cells passages 18–19, FPLD2 cells passages 20–23, MADB cells passages 16–17) control, HGPS, FPLD2, and MADB fibroblasts. Cell strains used for control (GM05567A, GM05757C, GM01651C), HGPS (HGADFN003, HGADFN164, HGADFN178), FPLD2 (CCLMA00336s, CCLMS337s, CCBB00466s), and MADB (PSADFN317, PSADFN318) fibroblasts. Cells were counterstained with DAPI. Scale bar 100 μm. (**b**) Quantification of the number of prelamin A positive nuclei in young (SNS ≤ 5%) and old (SNS ≥ 20%) control, HGPS, FPLD2, and MADB fibroblasts. (**c**) Representative images of normal (ovoid) and dysmorphic nuclei (abnormal and/or large nuclear shape), counterstained with DAPI. Scale bar 50 μm. (**d**) Quantification of the number of dysmorphic nuclei in young (SNS ≤ 5%) and old (SNS ≥ 20%) control, HGPS, FPLD2, and MADB fibroblasts. (**b**,**d**) Values are presented as mean ± SD (*n* = 3); not significant (ns); * *p* < 0.05; ** *p* < 0.01; *** *p* < 0.001; **** *p* < 0.0001; unpaired *t*-test.

Prelamin A and progerin were not detected in young control fibroblast cultures, whereas 10% of the cells were prelamin A-positive in old control cultures (Figure 6). In young and old HGPS fibroblast cultures, progerin was detected, and a weak signal for prelamin A was observed in brightly labeled progerin-positive cells (Figure 6). Hence, 27% of HGPS nuclei exhibited positive signal for prelamin A in young cultures, and this number increased to an average of 37% in later passages (Figure 6a,b). In FPLD2 and MADB cultures, although progerin was not detected, prelamin A was detected. Specifically, 18% of FPLD2 nuclei showed a weak prelamin A positive signal in young cultures but increased to 45% in late passages (Figure 6). In MADB fibroblast cultures, a strong prelamin A signal was detected in all nuclei from young and old passages, whereas progerin was not detected (Figure 6a,b). Furthermore, we scored the number of dysmorphic nuclei, showing abnormal and large nuclear morphologies instead of the typical ovoid nuclear shape, in fibroblast cultures from these three genetic disorders (Figure 6). In MADB, 35% of the nuclei were dysmorphic in early passages (<5% senescence), 19.6% in HGPS, and 16.7% in FPLD2 at similar passages (young cultures, Figure 6). In contrast the number of dysmorphic nuclei increased in old fibroblast cultures (senescence > 20%) from all three diseases including normal fibroblast cultures (Figure 6).

**Figure 7:**
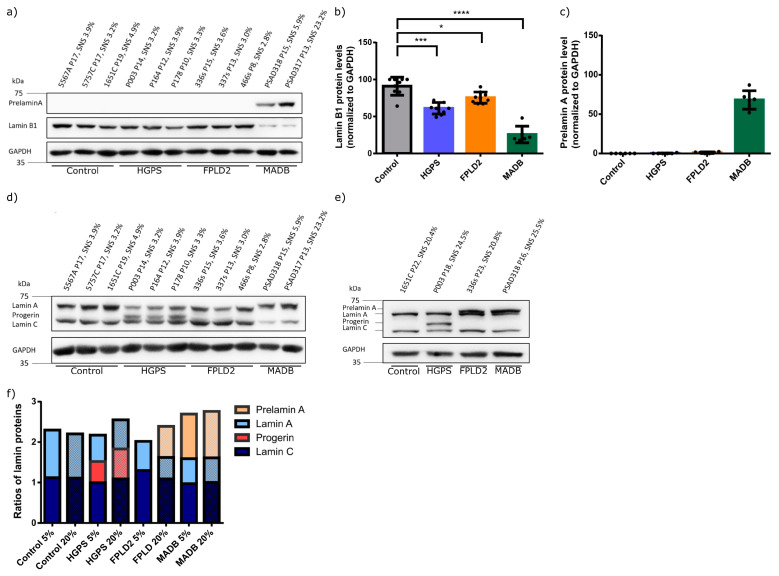
Progerin, prelamin A, lamin A/C, and lamin B1 status in young (SNS ≤ 5%, control cells passages 16–21, HGPS cells passages 10–14, FPLD2 cells passages 9–14, MADB cells passages 12–14) and old (SNS ≥ 20%, control cells passages 28–31, HGPS cells passages 18–19, FPLD2 cells passages 20–23, MADB cells passages 16–17) fibroblast cultures from different laminopathies associated with lipodystrophy. (**a**) Representative image of western blots for prelamin A and lamin B1 (*n* = 3). The percentage of senescence (SNS) cells in the cultures is indicated. (**b**) Quantification of lamin B1 levels. (**c**) Quantification of prelamin A levels. (**d**,**e**) Representative images of western blots for lamin A/C, prelamin A, and progerin in young and old fibroblast extracts (*n* = 3). (**f**) Ratio of prelamin A, lamin A, progerin, and lamin C in young and old fibroblasts (*n* = 3). (**b**,**c**) Graph show mean ± SD (*n* = 3); not significant (ns); * *p* < 0.05; *** *p* < 0.001; **** *p* < 0.0001; unpaired *t*-test.

Western blot analyses were performed to quantify the levels of progerin, prelamin A, lamin B1, and lamin A/C expression levels in total protein from young (SNS ≤ 5%) and old (SNS ≥ 20%) fibroblast cultures. Lamin B1, like Lamin A/C, plays an important role in the build-up of the nuclear lamina structure and integrity and participates in chromatin and genome organization [65]. Lamin B1 expression was significantly lower in all three laminopathies, with HGPS and MADB cells having the lowest expression levels (Figure 7a,b). Compared with the control, there was a decrease in Lamin B1 by 30% in HGPS, 15% in FPLD2, and 60% in MADB (Figure 7b), which was confirmed by immunocytochemistry (Figure S1).

Expectedly, progerin was detected in both young and old HGPS fibroblasts (Figure 7d,e). In FPLD2 cells, prelamin A was detected only in old fibroblast cultures (Figure 7a–e). MADB cells showed high levels of prelamin A in young and old cells and low levels of lamin A (Figure 7c–f). In young and old control cells, lamin A/C signals were detected, but no progerin or prelamin A signals were detected (Figure 7d–f). Compared to control fibroblasts, lamin A expression level was lower in all laminopathies (Figure 7f).

Collectively, the expression of progerin or prelamin A in fibroblasts derived from these three laminopathies-caused alterations in A-type lamin proportions and, in addition, in lamin B1. These alterations are responsible for the perturbation of the lamina composition, which consequently induces nuclear envelope abnormalities, as indicated by the increased incidence of dysmorphic nuclei.

## 4. Discussion

In patients with HGPS, lipodystrophy is one of the main symptoms that can appear as early as at six months of age [23]. An alteration in adipocyte tissue ratio has a far-reaching effect on body functions and health status and is associated with autoimmune and cardiovascular diseases [22]. Therapies targeting lipodystrophy remain poorly explored, indicating the need for further studies, especially for cases associated with laminopathies. Presently, lonafarnib (FTI) is the only FDA-approved treatment for HGPS [48]. FTI has been reported to improve the HGPS cellular phenotype, ameliorate the cardiovascular burden, increase bone mineral density, and extend the life expectancy [1,46,47,48,66,67]. Nevertheless, it is associated with cellular side effects, such as donut-shaped nuclei, mitotic errors, genomic instability, anti-proliferative effects, and blockade of the farnesylation of functional proteins other than prelamin A [44,49,50,68,69,70,71,72,73,74].

In the present study, FTI treatment was ineffective in promoting adipogenesis in SKPs derived from normal, HGPS, MADB, and FPLD2 individuals. However, FTI-treated normal SKPs showed a comparable extent of adipocyte differentiation as their mock-treated counterparts, indicating that targeting the prenylation of prelamin A, progerin, and other prenylated proteins is not essential for adipogenesis. This suggest that FTI does not directly affect the signaling pathways or transcription factors responsible for the regulation of adipocyte differentiation [75]. In contrast, other studies have shown that FTI treatment can inhibit adipogenesis by interfering with adipogenic pathways and reducing the expression of the peroxisome proliferator-activator receptor γ (PPARγ) and CCAAT/enhancer binding protein α (C/EBPα), which are key transcription factors involved in adipogenesis [76]. Additionally, FTIs have been shown to inhibit the PI3K/Akt pathway by interfering with the prenylation and activation of small GTPases, such as Rho, Rac, and Cdc42, which are involved in the activation of PI3K [77,78]. Hence, inhibition of PI3K/Akt results in inactivation of its downstream target mTOR, inducing a decrease in protein synthesis and the expression of PPARγ and C/EBPα [79]. However, FTI directly blocks the prenylation of Rheb, an activator of mTOR, and has similar effects on the levels of these adipogenic transcription factors [80]. Furthermore, FTI may interfere with adipogenesis through antiproliferative and apoptotic effects via increasing ROS levels [81]. Specifically, FTI can interfere with adipogenesis in cancer cells, leading to oxidative DNA damage [82,83]. Under normal conditions, low ROS levels are necessary for adipocyte differentiation, whereas high ROS levels have a negative impact [84]. However, despite mild drug-related side effects such as diarrhea, fatigue, nausea, vomiting, and anorexia, FTI is well tolerated and safe for children with HGPS [85]. To overcome the limitation of FTI, the identification of novel compounds that can ameliorate lipodystrophy and are compatible with FTI is necessary. Therefore, we examined whether Bar, an FDA-approved JAK/STAT inhibitor, can ameliorate adipogenesis in HGPS-SKPs and exert a synergistic effect in combination with FTI. Treatment with Bar alone and in combination with FTI improved adipocyte differentiation and lipid droplet formation in cells derived from patients with three distinct diseases characterized by lipodystrophy. Although HGPS, FPLD2, and MADB have different molecular mechanisms, they all share a common etiology, which is the accumulation of abnormal lamin A [13].

Lamin A plays an important role in adipogenesis and normal cell function, and the toxic accumulation of progerin or farnesylated prelamin A causes oxidative stress and mitochondrial dysfunction, driving premature senescence [86,87,88,89]. Although FPLD2 is associated with mutations in LMNA that do not directly cause prelamin A accumulation, cellular age-dependent farnesylated prelamin A accumulation has been observed in fibroblasts from these patients. In present studies, the accumulation of progerin in HGPS and prelamin A in these three distinct diseases contributed to defects in adipogenesis. A previous study showed that treatment with Bar alone or in combination with FTI can improve HGPS cellular homeostasis and delay senescence [49]. Additionally, Bar treatment induced inhibition of the JAK/STAT signaling, enhanced progerin clearance, ameliorated the nuclear shape, decreased SASP, and delayed senescence [49]. The JAK/STAT pathway is overactivated in HGPS fibroblasts, triggering chronic inflammation and the secretion of pro-inflammatory factors [50]. Moreover, previous studies have shown that high levels of pro-inflammatory factors, such as IL-6, TGFβ, and TNF, promote cells to senescence and negatively affect adipogenesis [84]. Importantly, Bar treatment significantly inhibited JAK/STAT signaling in fibroblasts, thereby reducing the levels of pro-inflammatory markers [49,50]. Since senescent cells secrete SASPs, which include pro-inflammatory factors, an increase in their expression negatively affects adipogenesis [58]. Similarly, the presence of high number of senescent cells dramatically reduced the adipocyte differentiation potential of SKPs, whereas Bar treatment decreased the number of senescent cells and improved adipocyte differentiation [58]. In this present study, treatment with Bar and Bar + FTI ameliorated adipogenesis and lipid droplet formation. However, Bar + FTI treatment showed no additive effects relative to the Bar treatment alone, indicating that the beneficial effect of Bar was maintained in the presence of FTI, and that the combination is not toxic to the cells.

Patients with HGPS exhibit several cellular and tissue defects, including lipodystrophy, and FTI treatment alone is ineffective against all these symptoms. Therefore, we hypothesized that the combined FTI and Bar by targeting different cellular processes would further benefit patients with HGPS. Expectedly, Bar + FTI treatment improved adipocyte differentiation in SKPs derived from patients with FPLD2 and MADB. However, in MADB cells, the size of the lipid droplets was similar to that observed in normal adipocytes, in contrast to HGPS and FPLD adipocytes. Studies on ZMPSTE24-deficient mouse models have shown that fatty acid, glucose, and triglyceride levels are similar to those in wild-type mice [88,90]. Long chain fatty acids, such as triglycerides, accumulate in adipocytes to form lipid droplets [21]. The normal size of lipid droplets observed in MADB cells might suggests that ZMPSTE24 mutations may not severely affect lipogenesis; however, this requires further investigation.

The molecular mechanisms underlying HGPS-, FPLD2-, and MADB-associated lipodystrophy are likely different because these three diseases are linked to different mutations. HGPS and MADB disorders are linked to premature aging and lipodystrophy, while FPLD2 is mainly associated with alterations in adipogenesis with partial fat accumulation and metabolic syndrome [1,13,22,26]. To understand how lipodystrophy occurs in these three distinct genetic disorders, we examined the mechanism through which they alter adipogenesis. In this study, HGPS fibroblasts accumulated progerin and low levels of prelamin A; FPLD2 fibroblasts also accumulated low levels of prelamin A, while MADB fibroblasts constitutively expressed prelamin A due to mutation in ZMSPTE24. Consequently, all three cell models accumulated farnesylated prelamin A or progerin [26]. Overall, cells derived from these pathologies exhibited dysmorphic nuclei, nuclear blebbing, cellular senescence, and low proliferation rate [12,35,91]. However, the cellular alterations were more severe in HGPS and MADB cells than in FPLD2 cells, reinforcing the hypothesis that farnesylated prelamin A or progerin are critically toxic to cells. Accumulation of farnesylated prelamin A or progerin lead to DNA damage, altered chromatin organization, and changes in gene expression [49,92,93,94,95].

Previous studies have shown that the accumulation of prenylated prelamin A isoforms is concomitantly followed by a reduction in lamin B1 [96,97]. Similarly, there was a decreased in lamin B1 levels in HGPS and FPLD2 fibroblasts in the present study, and this decrease was more prominent in MADB cells. A reduction in lamin B1 levels is linked to cellular senescence and changes in the lamina composition known to affect chromatin arrangement, replication, and transcription [98]. Hence, the lamina structure plays a role in mechanosensing, with lamin A and C providing nuclear stiffness and lamin B contributing to elasticity and deformation of the nuclear envelope [99]. Mutations in LMNA or ZMSPTE24 affect the composition of the lamina and can consequently alter the mechanotransduction of the nucleus and its response to intra- and extracellular signals [100,101,102]. Although it remains unclear why LMNA mutations affect lamin B1 levels, DNA damage and cellular senescence appear to be associated with reduced lamin B1 levels [96,99]. High levels of lamin B1 are expressed in preadipocytes and adipocytes [103], and its loss may contribute to alterations in nuclear membrane permeability and function [104,105]. Collectively, alterations in the nuclear lamina composition of HGPS, FPLD2, and MADB cells may underly the adipogenesis defects observed in these three pathologies.

In the present study, we demonstrated the beneficial effect of Bar treatment alone and Bar+ FTI treatment on adipogenesis in HGPS, FPLD2, and MADB SKPs. Although in vivo studies are necessary to validate these results, our findings suggests that the Bar + FTI treatment combination might have therapeutic benefits for patients with HGPS-, FPLD2-, and MADB-associated lipodystrophy and possibly other age-related diseases.

## Data Availability

Data are contained within the article and Supplementary Materials.

## Supplementary Materials

The following supporting information can be downloaded at: https://www.mdpi.com/article/10.3390/cells12101350/s1, Figure S1: Lamin B1 detection in HGPS, FPLD2, and MADB fibroblasts; Figure S2: Full-length scan of western blots of Figure 7.
