# Supplementary material for: Impact of Combined Baricitinib and FTI Treatment on Adipogenesis in Hutchinson–Gilford Progeria Syndrome and Other Lipodystrophic Laminopathies
Source: Cells. 2023 May 9;12(10):1350. doi: 10.3390/cells12101350 (PMC10216179; doi:10.3390/cells12101350)
Supplement: Supplementary file 1 [file cells-12-01350-s001.zip › cells-2354119-supplementary.pdf]

Supplementary Figures

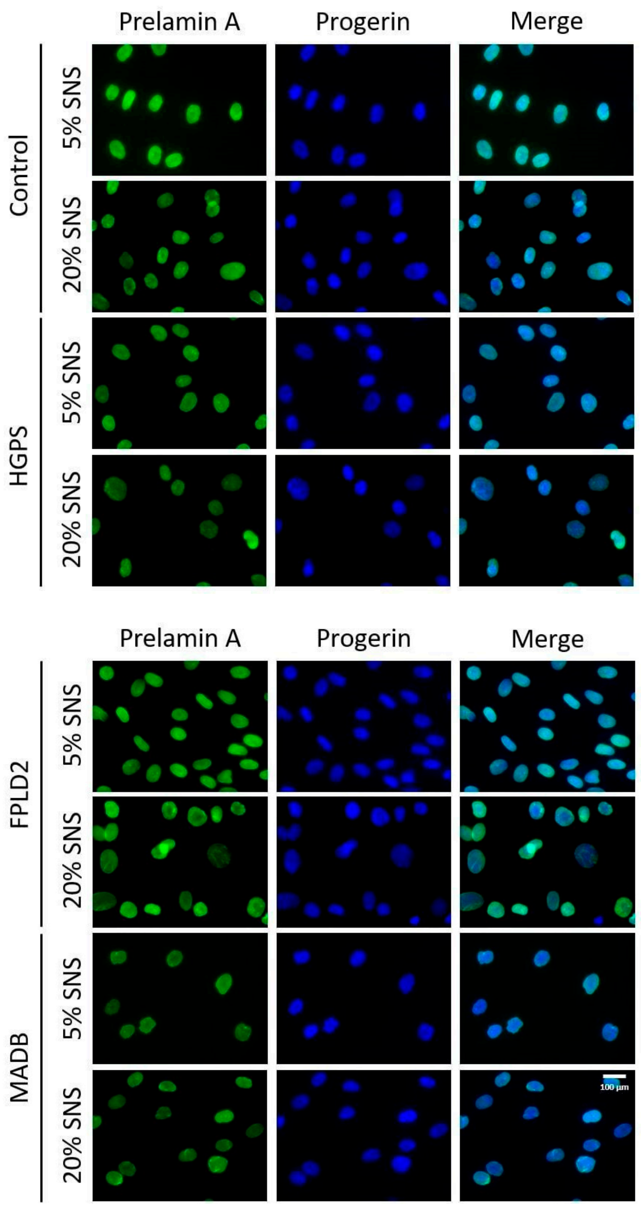

Figure S1. Lamin B1 detection in HGPS, FPLD2, and MADB fibroblasts. Immunohistochemistry for lamin B1 in young (SNS ≤5%, controll passages 16-21, HGPS passages 10-14, FPLD2 passages 9-14, MADB passages 12-14) and old (SNS ≥20%, controll passages 28-31, HGPS passages 18-19, FPLD2 passages 20-23, MADB passages 16-17) control, HGPS, FPLD2 and MADB fibroblasts. Cells were counterstained with DAPI. Scale bar 100 µm.

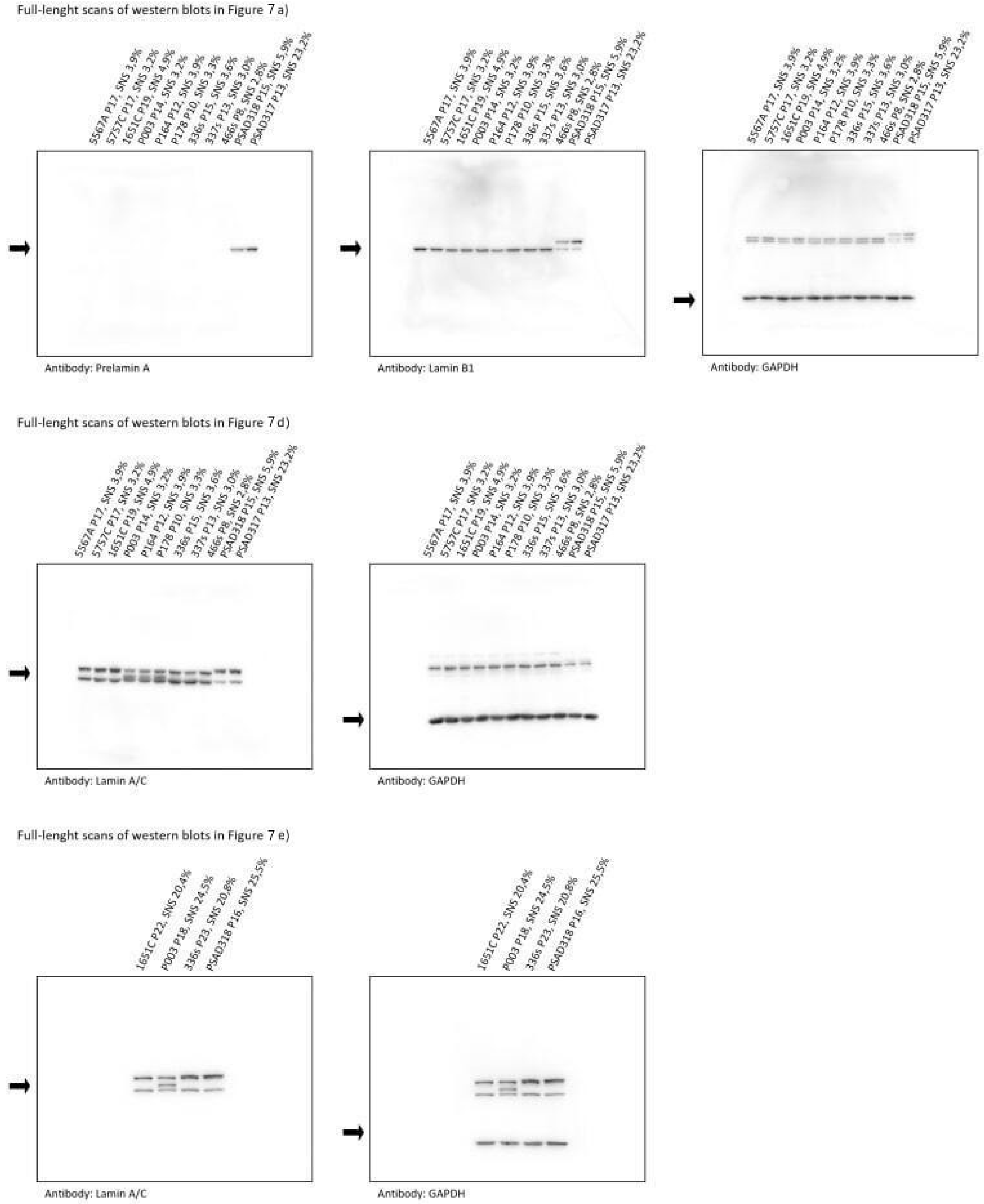

Figure S2. Full-length scan of western blots from Figure 7a, d, e.
